# Analysis on the factors associated with COVID-19 infection among Chinese residents after the implementation of *the 10 new rules* to optimize COVID-19 response: a cross-sectional study

**DOI:** 10.3389/fpubh.2023.1197889

**Published:** 2023-06-08

**Authors:** Yunshu Li, Sunyi Wang, Nan Yang, Yuwen Shi, Yingxiao Yang, Zhixin Zhu, Xiuyang Li

**Affiliations:** ^1^Department of Big Data in Health Science and Center for Clinical Big Data and Statistics, Second Affiliated Hospital, Zhejiang University College of Medicine, Hangzhou, China; ^2^School of Public Health, College of Medicine, Zhejiang University, Hangzhou, China

**Keywords:** COVID-19 infection, regular epidemic prevention and control, related factors, logistic regression, policy analysis

## Abstract

**Introduction:**

This study aimed to investigate the status of COVID-19 infection and the associated factors among Chinese residents after the implementation of *the 10 New Rules* to optimize COVID response.

**Methods:**

Participants were recruited using convenience sampling. The study used self-filled questionnaires to examine COVID-19 infection and associated factors among Chinese residents, from December 29, 2022, to January 2, 2023. For the statistical analysis, descriptive and quantitative analyses were used. The potential risk factors for COVID-19 infection were identified by multivariable logistic regression analysis.

**Results:**

After the adjustments in control strategies against COVID-19, the infection rate of COVID-19 was high among respondents, and 98.4% of individuals who tested positive showed symptoms including cough, fever, fatigue, headache, sore throat, nasal congestion, sputum production, muscle and joint pain, and runny nose. The main problems respondents reported were the shortage of drugs and medical supplies, the increased burden on families, and the unreliable information source of COVID-19 infection. Logistic regression showed that isolating patients with COVID-19 at home was associated with a lower risk of COVID-19 infection (OR = 0.58, 95%CI: 0.42–0.81).

**Conclusion:**

COVID-19 infection among residents is closely related to age, gender, and epidemic prevention measures. The government needs to strengthen education for individuals and centrally manage and properly address difficulties that may arise during COVID-19.

## Introduction

Since the COVID-19 epidemic origination in Hubei in early 2020, China has adhered to its dynamic zero-COVID policy and strategies to tackle both imported and domestic infections. Nonpharmacological interventions were rapidly deployed to control COVID-19 outbreaks including epidemiological investigation, large-scale nucleic acid testings and strict lockdowns. People were urged to wear face masks, maintain personal hygiene, and maintain social distancing. Since February, 2022, mass vaccination was carried out throughout the country. By November 3, 2022, 31 provinces (autonomous regions and municipalities directly under the Central government) and Xinjiang Production and Construction Corps had reported receiving 344,0119 doses of COVID-19 vaccine. The above preventive measures were proved effective by research (1–5) and thanks to the dynamic zero-COVID policy, a low infection rate among the general population was maintained throughout the pandemic.

However, given the weakening pathogenicity of the Omicron variant, the dominant variant in China since early 2020, a high vaccination rate among the population, and the need to effectively coordinate the epidemic response with economic and social development, the Chinese government implemented a massive policy shift from the dynamic zero-COVID policy. On December7, 2022, the Joint Prevention and Control Mechanism of the State Council issued *the 10 new rules* (6) (shown in Supplementary material) to further optimize the prevention and control work of COVID-19 in China, which was the second adjustment after the implementation of *20 Optimization rules* (7). *The 10 new rules* were put into effect among the country on the very day. According to *the 10 new rules*, Relevant departments in localities were required to rectify oversimplified or one-size-fits-all approaches and excessive policy steps, and faithfully implement prevention and control measures to maximize the protection of people’s lives and health and minimize the impact of the epidemic on economic and social development. For example, COVID-19 risk areas were delineated in a science-based and targeted manner instead of expanding arbitrarily and people were no longer subjected to mass testing according to administrative regions and were no longer required to provide negative nucleic acid test results or undergo health code checks to access public venues or travel to other regions.

With China’s COVID-19 response optimization, there are rising concerns about how it affect the situation of COVID-19 infection among Chinese residents and what are the effective preventive measures for the residents. Therefore, we investigated the public health situation after the promulgation of the *10 New Rules*, survey questions including demographic data, vaccination situation, other preventive measures, whether infected with COVID-19, time of COVID-19 infection, symptoms, infection of cohabitants, drug reserves at home, and so on. We further analyzed the risk factors and protective factors of COVID-19 infection to provide a reference for efficient epidemic prevention and control in the new stage.

## Materials and methods

### Data source and study population

Respondents were recruited using convenience sampling techniques. Inclusion criteria: (1) Chinese residents; (2) Have the ability to complete the online questionnaire independently under the principle of informed consent. The online questionnaire was imported into an online survey platform (“SurveyStar,” Changsha Ranxing Science and Technology, Shanghai, China) and the QR code and the link of the questionnaire were then published on WeChat, alumni online forums, and other social media platforms. The questionnaires were filled in anonymously with the informed consent of the respondents. From December 29, 2022, to January 2, 2023, we received 1,016 online questionnaires with an effective rate of 99.11%.

### Questionnaire

The questionnaire used in this study was adapted from the second round of COVID-19 Infection Questionnaire released by China’s National Center for Disease Control and Prevention and Peking Union Medical College. The questionnaire was composed of 27 questions and covered several areas including demographic data, symptoms, and precautionary measures against COVID-19, shown in Supplementary material.

### Quality control

IP addresses were not allowed to fill in the questionnaire repeatedly. Questionnaires from respondents aged less than 1 year, blank questionnaires, questionnaires completed within 90 s, and questionnaires with the same option for all questions were considered invalid.

### Statistical analysis

Data were analyzed with SAS software version 9.4. Cronbach’s α and factor analysis were used to test the reliability and validity of the questionnaire, respectively. Univariate analysis and logistic regression analysis were performed to determine the influencing factors of COVID-19 infection. In univariate analysis, the chi-square test was used for count data, and the Wilcoxon rank sum test was used for grade data and measurement data. The statistically significant variables in the univariate analysis were included in logistic regression as independent variables. Enter method was used to deal with variables in logistic regression. Those who had tested positive or had typical COVID-19 symptoms were defined as infected respondents with COVID-19 and those who always tested negative or those not tested with no symptoms were defined as uninfected respondents. The significance level was set at *P* ≤ 0.1 for univariate analysis and was set at *P* ≤ 0.05 for logistic regression analysis.

## Results

In this study, we posted a QR code and link to the questionnaire online and received 1,016 questionnaires, 99.11% of them effective.

### Reliability and validity test

Cronbach’s α of the symptom dimension (question 11 in Supplementary questionnaire) was 0.854 (>0.7), indicating the internal consistency of the questionnaire was high.

The KMO value is 0.879 (>0.6), *P* < 0.001 Bartlett sphericity test sig = 0.000 < 0.001, indicating that factor analysis is suitable. The principal component method was used to extract the factors, and the maximum variance method was used to rotate the factors. After the absolute value of the coefficient was set to 0.5, three factors were extracted, and the cumulative variance interpretation rate was 51.87%. The symptoms belonged to component 1 (symptom), and the variance interpretation rate was 32.63%. The items income and education belong to component 2 (education level and income), and the variance interpretation rate was 10.11%. Hand washing and wearing a mask belong to component 3 (protective measures), and the variance interpretation rate is 9.12%. The load of all items was higher than 0.5 and all items passed the validity test.

### Basic characteristic

Most respondents were females (65.94%), were under 50 years old (84.91%), were undergraduates (55.41%), lived in Zhejiang province (82.72%), and had an annual household income per capita of more than 50,000 yuan (77.06%).

Since the publication of *the 10 New Rules*, 16.68% of the participants always tested negative for COVID-19, 27.01% were positive for the first time, 0.60% were re-positive, and 21.95% had tested positive but had turned negative. 33.76% did not undergo antigen tests or nucleic acid tests, of which 19.16% reported symptoms and 14.60% were asymptomatic. Meanwhile, for the current or past positive respondents, only 1.60% were asymptomatic, and the other patients reported symptoms mainly including cough, fever, fatigue, headache, sore throat, nasal congestion, sputum production, muscle and joint pain, and runny nose. Among the respondents, the vaccination rate reached 94.74%. The implementation rate for each of the three protective measures, i.e., scientific hand washing (the definition shown in Supplementary questionnaire), wearing masks, and isolating patients with COVID-19 at home was over 50% and the rate of medication use reached 66.83%. The main difficulties they were confronted with included difficulty in obtaining medications and medical equipment, the high burden of family care, difficulty in setting up an isolation environment, and so forth. Table 1 gives the characteristics of the respondents.

**Table 1:** Basic information of the respondents.

Variables		Case, n	Proportion, %
Gender	Male	343	34.06
	Female	664	65.94
Age	<30	297	29.49
	30–39	182	18.07
	40–49	376	37.34
	50–	152	15.09
Residence	Zhejiang	833	82.72
	Guangdong	24	2.38
	Shanghai	20	1.99
	Jiangsu	18	1.79
	Beijing	13	1.29
	Other	99	9.83
Education	Junior high school and below	74	7.34
	High school/vocational high school/junior high school	129	12.81
	Junior college degree	171	16.98
	Undergraduate	558	55.41
	Graduate and above	75	7.45
Annual household income per capita	¥ ≤ 30,000 yuan	87	8.64
	30,000 yuan<¥ ≤ 50,000 yuan	144	14.30
	50,000 yuan<¥ ≤ 100,000 yuan	234	23.24
	100,000 yuan<¥ ≤ 200,000 yuan	304	30.19
	¥ > 200,000 yuan	238	23.63
With a health-related major or occupation	Yes	134	13.31
	No	873	86.69
Status of COVID-19 infection	Antigen or nucleic acid detection	667	66.24
	Not yet infected, always negative	168	16.68
	Had tested positive, now turning negative	221	21.95
	First tested positive	272	27.01
	Re-positive in the second infection test	6	0.60
	Non-antigen or non-nucleic acid detection	340	33.76
	Significant symptoms, not tested	193	19.16
	No obvious symptoms, not tested	147	14.60
Symptoms of COVID-19	No symptoms	8	1.60
	Cough	411	82.36
	Fever	392	78.56
	Fatigue	339	67.94
	Headache	298	59.72
	Sore throat	295	59.12
	Nasal congestion	295	59.12
	Sputum production	285	57.11
	Muscle and joint pain	279	55.91
	Runny nose	253	50.70
	Altered sense of smell and taste	151	30.26
	Diarrhea	82	16.43
	Breath-holding, shortness of breath	63	12.63
	Vomiting	51	10.22
Vaccination status	No vaccination	53	5.26
	One dose of vaccination	16	1.59
	Two doses of vaccination	174	17.28
	Three doses of vaccination	721	71.60
	Four doses of vaccination	43	4.27
Manufacturer of the vaccine	Sinovac-CoronaVac COVID-19 vaccine	701	73.48
	Sinopharm COVID-19 vaccine (Beijing)	252	26.42
	Zhifei Longcom, China	48	5.03
	Sinopharm COVID-19 vaccine (Wuhan)	38	3.98
	CanSinoBIO COVID-19 vaccine	15	1.57
	Other	51	5.35
Prevention measures	Scientific hand washing	685	68.02
	Wearing masks	972	96.52
	Isolation measures	747	74.18
Medication for preventive or therapeutic purposes	Western medicine	528	52.43
	Chinese patent medicine	275	27.31
	Chinese medicine	84	8.34
	Other	38	3.77
	No medication	334	33.17
Difficulties during the pandemic	Difficulty in obtaining medications and medical equipment	588	58.39
	High burden of caring for family members	422	41.91
	Isolation environments are difficult to build	419	41.61
	No reliable source of information on COVID-19 infections	395	39.23
	Difficulty in accessing medical treatment	386	38.33
	High pressure of work and study	352	34.96
	Increased financial burden	258	25.62
	Shortage of necessities	113	11.22
	Other	68	6.75

### Univariate analysis of COVID-19 infection

Chi-squared test was performed for count data and the Wilcoxon rank sum test was performed for ranked data and measurement data to explore the association between sample characteristics and COVID-19 infection. Among the variables, gender, isolating patients with COVID-19 at home and age had a significant effect on COVID-19 infection. The infection rate of females was higher than that of males (*P* < 0.1). Those who did not isolate patients with COVID-19 at home were more likely to be infected than those who did (*P* < 0.1). And individuals infected with COVID-19 were younger than those uninfected (*P* < 0.1). The results are shown in Table 2.

**Table 2:** Univariate analysis of COVID-19 infection.^a^

Variables		Uninfected	Infected	Statistics	*P*
Gender	Male	120	223	3.32	0.068
	Female	195	469		
Age^b^		43(24)	39(24)	166455.50	0.072
Education level	Junior high school degree and below	32	42	6.75	0.149
	High school/vocational high school/technical secondary school degree	43	86		
	Junior college degree	51	120		
	Bachelor’s degree	170	388		
	Master’s degree and above	19	56		
Major of study or occupation related to healthcare	No	276	597	0.34	0.559
	Yes	39	95		
Vaccinated	No	18	35	0.19	0.665
	Yes	297	657		
Having received 2 or more doses	No	24	45	0.42	0.516
	Yes	291	647		
Vaccine manufacturer	Each dose provided by Sinopharm (Beijing)	190	412	0.25	0.884
	Each dose provided by Sinovac	52	110		
	Other	73	170		
Having proper hand-washing habits	No	101	221	0.00	0.968
	Yes	214	471		
Having the habit of wearing a mask	No	14	21	1.28	0.257
	Yes	301	671		
Isolating patients with COVID-19 at home	No	60	200	10.98	<0.001
	Yes	255	492		
Number of vaccine doses received	Unvaccinated	18	35	1.33	0.857
	1 dose	6	10		
	2 doses	49	125		
	3 doses	228	493		
	4 doses	14	29		
Annual household income per capita^c^	¥ ≤ 30,000 yuan	31	56	164305.00	0.178
	30,000 yuan < ¥ ≤ 50,000 yuan	35	109		
	50,000 yuan < ¥ ≤ 100,000 yuan	74	160		
	100,000 yuan < ¥ ≤ 200,000 yuan	95	209		
	¥ > 200,000 yuan	80	158		

^a^Count data were presented as numbers, and the chi-squared test was performed for comparison between groups.

^b^Measurement data were presented as medians with interquartile ranges (IQRs), and comparison between groups was performed by the Wilcoxon rank sum test.

^c^Ranked data were presented as numbers, and the Wilcoxon rank sum test was performed for comparison between groups.

### Multivariable logistic regression analysis of COVID-19 infection

Statistically significant variables in univariate analysis were screened and included in multivariate logistic regression analysis to determine potential risk factors for COVID-19 infection. Enter method was used to deal with variables in logistic regression. The analysis showed that isolating patients with COVID-19 at home was associated with a lower risk of COVID-19 infection (OR = 0.58, 95%CI: 0.42–0.81). The results are shown in Table 3.

**Table 3:** Multivariable logistic regression analysis of COVID-19 infection.

Variables		*β*	SE	Wald χ^2^	*P*	OR	95% CI
Gender	Male	Ref				1.00	
	Female	0.27	0.15	3.40	0.065	1.30	(0.98, 1.73)
Age	<30 years	Ref				1.00	
	30–39 years	0.29	0.22	1.75	0.186	1.33	(0.87, 2.04)
	40–49 years	−0.29	0.17	2.89	0.089	0.75	(0.54, 1.05)
	≥50 years	−0.13	0.22	0.37	0.541	0.88	(0.57, 1.34)
Isolating patients with COVID-19 at home	No	Ref				1.00	
	Yes	−0.54	0.17	10.38	0.001	0.58	(0.42, 0.81)

## Discussion

In our study, we found that since the adjustment of the prevention and control policy of *the 10 New Rules China*, the transmission rate of COVID-19 infection in China has been much faster than in the past few years, and the infection rate of Chinese residents has increased rapidly. This is consistent with the trend of the positive number and positive rate of nucleic acid testing of novel coronavirus reported by the Chinese Center for Disease Control and Prevention. As of 24:00 p.m. on January 2, more than half of the respondents tested positive or had significant symptoms. Among the respondents who tested positive, the asymptomatic patients accounted for only 1.6%, indicating that most of the infected people would have varying degrees of symptoms, which contradicts the findings in Oran’s study (8) that at least one-third of SARS-CoV-2 infected people were asymptomatic and nearly three-quarters of people who were positive for PCR would remain asymptomatic throughout the disease. Evidence showed that the varying degrees of symptoms, ranging from no symptoms (asymptomatic) to severe symptoms were due to organism differences (9–11) while the increased proportion of asymptomatic infection may be related to the mutation of the novel coronavirus strain with the increase of infected people (12).

At the same time, the survey population was mainly in Zhejiang Province, thus the proportion of asymptomatic patients may not represent the national level as the mobility of the population was low during the winter holiday, and the temperature and economic level among provinces differed (13, 14). Researches have found that most asymptomatic infected people did not seek medical treatment due to their lack of obvious clinical symptoms and awareness of prevention, leading to the rapid spread of the COVID (15). Therefore, the assessment and management of this specific type of infection is a huge challenge that is worth more attention. Since asymptomatic individuals can transmit the virus and the long-term effects are unclear, residents should take personal protection and seek medical help if necessary.

Among our respondents, common clinical characteristics were cough, fever, fatigue, headache, sore throat, nasal congestion, and sputum production, which was consistent with the conclusion in *the Tenth Edition of Diagnosis and Treatment Protocol for COVID-19* (16). The most common symptom in our study was “cough,” while in many studies “fever” was the most common clinical symptom (17–19). It is speculated that the difference may be related to the dominant COVID-19 strain circulating in China, the production of antibodies in the body after vaccination, environmental temperature, and other factors. The Omicron variant appeared in November 2021. Its transmission and immune escape ability were significantly enhanced and in early 2022, the Omicron variant quickly replaced the Delta variant as the dominant epidemic strain globally. Our study showed that the pulmonary pathogenicity of the Omicron variant had significantly weakened, and the most common clinical manifestation had changed from pneumonia to upper respiratory tract infection (16). As a result, China’s testing methods, clinical treatment and social control strategies for the novel coronavirus have also changed, from centralized nucleic acid testing to recommended antigen self-testing, no longer distinguishing between secondary contact, and reducing the length of quarantine for inbound travelers.

Univariate analysis of infection showed that the infection rate was higher in females than in males. This is consistent with the results of the first COVID-19 reports in China in 2019. Guan and Zhong pointed out gender imbalance in COVID-19 detection rates and fatality rates (20). After studying 6,013 confirmed cases, the team at Wuhan University (21) found that females accounted for a lower proportion and had milder symptoms at the early stage of the epidemic. However, in the late stage, the proportion of females increased and their symptoms worsened. This was possibly because the female had higher immunity thus their incubation period was longer. However, there is still a lack of thorough analysis on the mechanism of difference between male and female infection, and the gender difference shown in our study may also be affected by the gender distribution, regional distribution, and detection methods of the research population (22). Further research is required to understand the molecular and pathophysiological mechanisms underlying these gender differences in COVID-19 infection and severity. Comparison among age groups showed that the infection rate was lower among people over 50 years old, which is consistent with other studies (23). It may be related to the decline of immune function in aging individuals, which would result in less obvious symptoms and being classified as uninfected. Logistic regression of factors related to COVID-19 infection showed that infection was closely related to home isolation habits, and the infection rate was lower if quarantine measures were taken than if no quarantine measures were taken. Therefore, it is recommended that residents should stay at home, ventilate the room and disinfect surfaces to reduce the risk of infection and to protect family members.

There are still some limitations in our study: (1) This survey recruited participants through social media platforms, thus may be biased in the selection of the region, educational background, and occupation. (2) The questionnaire did not carry out a complete validity analysis, and all the data were filled in by respondents on the Internet. Although data review and quality control were carried out in the later period, there may still be information bias. (3) The survey lacks longitudinal follow-up, and thus cannot access the recovery, sequelae, and reinfection of COVID-19 which are worth further investigation. (4) This survey lacked laboratory and imaging data, and thus could not fully reflect the clinical characteristics of patients, and we did not investigate the respondents’ underlying diseases of the cardiovascular system, respiratory system, and endocrine system. (5) Considering that many residents used home-based antigen self-test test, we counted both antigen detection and nucleic acid detection as diagnostic criteria in our study. However, the sensitivity and specificity of antigen detection were not high, thus it could lead to miss diagnosis and false positives.

This study is one of the first group of studies to investigate the infection situation of Chinese residents after the implementation of *the 10 New Rules*. The results can reflect the real situation at that time, alleviate the panic and concern of Chinese people to a certain extent, and provide practical evidence and inference for the implementation of public health policies in the future. Through this survey, we found that with the further relaxation of national prevention and control measures, every resident’s risk of contracting the novel coronavirus has also increased. Epidemiological data (24) show that the number of COVID-19 cases increases exponentially in the early stage, and the earlier the case is detected and relevant measures are taken, the easier the epidemic will be controlled.

Therefore, every resident needs to take quarantine measures to reduce the risk of infection. Also, learning relevant scientific knowledge in advance can help to protect ourselves and our families (25). At the social level, it is necessary to understand the difficulties and needs of residents during the COVID-19 pandemic, to provide timely support and assistance for such problems as “Difficulty in obtaining medications and medical equipment,” “High burden of caring for family members,” “difficulty in setting up an isolation environment” and “Lack of reliable source of information on COVID-19 infections,” and to alleviate temporary difficulties and inconveniences through materials allocation and personnel transportation. At the same time, all efforts should be made to ensure timely treatment for those infected with the novel coronavirus, to reduce the rate of severe illness as much as possible. With the concerted efforts of the whole society, the epidemic will eventually be overcome.

## Conclusion

Our study aimed to investigate the domestic situation of novel coronavirus infection and analyzed possible influencing factors related to infection after the implementation of *the 10 New Rules* China, to explore feasible policy measures for the development and control of the epidemic. The results showed that the infection rate of the population increased significantly within a very short time after China adjusted the control measures. It is necessary to do a good job in popular science education and treatment of critically infected patients, eliminating panic among the public, securing social resources, and effectively dealing with the epidemic prevention and control work in the new stage.

## Data availability statement

The raw data supporting the conclusions of this article will be made available by the authors, without undue reservation.

## Author contributions

YL, SW, NY, YS, and YY designed the study, acquired the data, and drafted the manuscript. YS designed the questionnaire. YL and NY designed the analysis plan and performed the statistical analyses. ZZ supervised the data analysis and interpretation. XL edited and proofread the manuscript. YL and SW had the primary responsibility for the study. All authors read and approved the published version of the final manuscript.

## Conflict of interest

The authors declare that the research was conducted in the absence of any commercial or financial relationships that could be construed as a potential conflict of interest.

## Publisher’s note

All claims expressed in this article are solely those of the authors and do not necessarily represent those of their affiliated organizations, or those of the publisher, the editors and the reviewers. Any product that may be evaluated in this article, or claim that may be made by its manufacturer, is not guaranteed or endorsed by the publisher.
