# Supplementary material for: Analysis on the factors associated with COVID-19 infection among Chinese residents after the implementation of the 10 new rules to optimize COVID-19 response: a cross-sectional study
Source: Front Public Health. 2023 Jun 8;11:1197889. doi: 10.3389/fpubh.2023.1197889 (PMC10285290; doi:10.3389/fpubh.2023.1197889)
Supplement: Supplementary file 1 [file Data_Sheet_1.docx]

Supplementary Material

**Supplementary Questionnaire**

**Questionnaire on COVID-19 infection after the Implementation of *the 10 New Rules***

**I.** **Personal information and vaccination status**

1. Your gender:

○Male

○Female

2. Your birth year:

_________________________________

3. Your geological location:

_________________________________

4. Your vaccination status:

○Having received one dose

○Having received Two doses

○Having received Three doses

○Having received Four doses

○Unvaccinated

5. Manufacturer of the vaccine you received: [Multiple-choice]

□ Sinopharm(Beijing)

□ Sinopharm(Wuhan)

□ Sinovac-CoronaVac

□ CanSinoBIO

□ Zhifei Longcom, China

□Other _________________

**II. COVID-19 infection**

6. Are you infected with COVID-19?

○I currently test negative.

○I currently test positive.

○I’m not tested but have typical symptoms(fever, dry cough, fatigue, abnormal smell and taste, etc.).

○I’m not tested and have no obvious symptoms.

7. Are you positive for the first time or re-positive?

○I’m positive for the first time.

○I’m re-positive.

[Dependent on the second choice of question 6]

8. Is the above test result made by antigen detection or nucleic acid detection?

○Nucleic acid test

○Antigen self-test

○Both

[Dependent on the first and second choice of question 6]

9. Have you ever tested positive?

○No, I’m always negative.

○Yes, I had tested positive, but I’m now negative.

[Dependent on the first choice of question 6]

10. The date of your first positive test:

_________________________________

11. Do you have any of the following symptoms: [Multiple-choice]

□ No symptoms

□ Fever _________________*

The maximum body temperature at the time of fever is?

□ fatigue

□ Headache

□ Muscle and joint pain

□ Sore throat

□ Nasal congestion

□ Runny nose

□ Cough

□ sputum production

□ Abnormal sense of smell and taste

□ Diarrhea

□ Vomiting

□ Breath-holding, shortness of breath

12. How long did your fever last:

○within 1 day

○1-2 days

○3-4 days

○5 days or more

13. Have you gone to hospital for COVID-19 infection since 11.11?

○Yes

○No

**III. Daily life**

14. Since the adjustment of the control measures for COVID-19, the main difficulties you have been confronted with are: [Multiple-choice]

□ Unreliable source of information on COVID-19 infection

□ Difficulty in obtaining medications and medical equipment (e.g.oximeters)

□ Shortage of necessities (food, clothing, etc.)

□ Difficulty in setting up an isolated environment

□ High burden of caring for family members

□ High pressure of work and study

□ Increased financial burden

□ Difficulty in accessing medical treatment

□ Other _________________

15. Do you have proper hand-washing habits?

To wash your hands properly, you need to meet four criteria: ① wash your hands before meals, after going to the toilet, after work, after touching money, after going to the hospital and after touching patients, etc.; ② wash your hands with running water; ③ use soap or hand sanitizer, etc. when washing your hands; ④ wash your hands for at least 20 seconds.

○Yes, I can meet the above four criteria in my daily hand washing

○No, I have not yet done so

16. Do you have the habit of wearing a mask?

○Yes

○No

17. How many people do you live with?

_________________________________

18. Are there any COVID-19 patients living with you?

○Yes

○No

19. Did you do you plan to isolate patients infected with COVID-19 at home?

○Yes

○No

20. Do you take medication for preventive or therapeutic purposes and what kind of medication do you choose? [Multiple-choice]

□ No medication

□ Western medicine (such as ibuprofen, Tylenol, Merlin, acetaminophen)

□ Chinese patent medicine

□ Chinese medicine

□ Other _________________

21. The preventive or therapeutic effect of the medication you took:

|  | Very effective | Effective | Unclear | Ineffective | Completely ineffective |
| --- | --- | --- | --- | --- | --- |
| Drug Effect | ○ | ○ | ○ | ○ | ○ |

**IV. General Information**

22. Your level of education or the degree you are currently pursuing:

○Junior high school degree and below

○High school/vocational high school/technical secondary school degree

○Junior college degree

○Bachelor's degree

○Master's degree and above

23. Are you currently studying in a college or university?

○Yes

○No

24. Your major of study:

_________________

25. Are you currently living on campus:

○Yes

○No

26. Is your major of study or occupation related to healthcare:

○Yes

○No

27. Your annual household income per capita (total household income in a year ÷ household size):

○¥≤30,000 yuan

○30,000 yuan <¥≤50,000 yuan

○50,000 yuan <¥≤100,000 yuan

○100,000 yuan <¥≤200,000 yuan

○¥ > 200,000 yuan

**Supplementary Document**

***The 10 new measures:***

1. Be precise in categorizing high-risk areas, limiting them to residential units or blocks and refraining from extending them at will to an entire housing compound, neighborhood or street.

2. Reduce mass testing frequency and further minimize its scale. Negative tests or a green health code on contact-tracing smartphone apps will no longer be required to access most public areas, excluding hospitals, senior homes, kindergartens. On-arrival testing and checking of health codes will end for domestic travel.

3. Asymptomatic and mild Covid cases (who satisfy quarantine conditions) can stay at home or voluntarily go to a centralized quarantine facility for seven days. Close contacts can quarantine at home for five days.

4. Lockdowns in high-risk areas will be lifted if there are no new cases for five consecutive days.

5. Ensure the availability of medicine for the public. Pharmacies cannot close at will, and no purchase limits for medicine will be allowed.

6. Accelerate vaccination for the elderly. Establish green lanes and mobile vaccination spots, improve publicity, and consider incentives to promote vaccine usage.

7. Enhance the monitoring of population still at risk for Covid.

8. the normal operations for key societal services. Low-risk areas are not allowed to control movement or suspend any services, work, or production. Public workers must promptly address the population's basic living needs.

9.Forbid the blockage of emergency exits or apartment entrances. Escape routes must stay open in the event of medical emergencies or hazards.

10. Improve pandemic control measures on school campuses. Schools without Covid cases must carry out offline learning and keep shared spaces open (e.g. cafeterias, libraries, stadiums). Schools with Covid cases must be precise in categorizing high-risk areas and ensure that low-risk areas continue as normal.
